# Connecting Replication and Repair: YoaA, a Helicase-Related Protein, Promotes Azidothymidine Tolerance through Association with Chi, an Accessory Clamp Loader Protein

**DOI:** 10.1371/journal.pgen.1005651

**Published:** 2015-11-06

**Authors:** Laura T. Brown, Vincent A. Sutera, Shen Zhou, Christopher S. Weitzel, Yisha Cheng, Susan T. Lovett

**Affiliations:** Department of Biology and Rosenstiel Basic Medical Sciences Research Center MS029, Brandeis University, Waltham, Massachusetts, United States of America; University of Michigan, UNITED STATES

## Abstract

Elongating DNA polymerases frequently encounter lesions or structures that impede progress and require repair before DNA replication can be completed. Therefore, directing repair factors to a blocked fork, without interfering with normal replication, is important for proper cell function, and it is a process that is not well understood. To study this process, we have employed the chain-terminating nucleoside analog, 3’ azidothymidine (AZT) and the *E*. *coli* genetic system, for which replication and repair factors have been well-defined. By using high-expression suppressor screens, we identified *yoaA*, encoding a putative helicase, and *holC*, encoding the Chi component of the replication clamp loader, as genes that promoted tolerance to AZT. YoaA is a putative Fe-S helicase in the XPD/RAD3 family for which orthologs can be found in most bacterial genomes; *E*. *coli* has a paralog to YoaA, DinG, which possesses 5’ to 3’ helicase activity and an Fe-S cluster essential to its activity. Mutants in *yoaA* are sensitive to AZT exposure; *dinG* mutations cause mild sensitivity to AZT and exacerbate the sensitivity of *yoaA* mutant strains. Suppression of AZT sensitivity by *holC* or *yoaA* was mutually codependent and we provide evidence here that YoaA and Chi physically interact. Interactions of Chi with single-strand DNA binding protein (SSB) and with Psi were required to aid AZT tolerance, as was the proofreading 3’ exonuclease, DnaQ. Our studies suggest that repair is coupled to blocked replication through these interactions. We hypothesize that SSB, through Chi, recruits the YoaA helicase to replication gaps and that unwinding of the nascent strand promotes repair and AZT excision. This recruitment prevents the toxicity of helicase activity and aids the handoff of repair with replication factors, ensuring timely repair and resumption of replication.

## Author Summary

During the replication of the cell’s genetic material, difficulties are often encountered. These problems require the recruitment of special proteins to repair DNA so that replication can be completed. The failure to do so causes cell death or deleterious changes to the cell’s genetic material. In humans, these genetic changes can promote cancer formation. Our study identifies a repair protein that is recruited to problem sites by interactions with the replication machinery. These interactions provide a means by which the cell can sense, respond to and repair damage that interferes with the completion of DNA replication.

## Introduction

All cells must balance ongoing DNA synthesis with repair reactions that are necessary to overcome problems in replication. Persistent, unreplicated single-strand gaps in DNA may be caused by lesions or by DNA structures that impede DNA polymerization in either the replication template or the nascent strand. Filling of single-strand DNA (ssDNA) gaps (a process termed “post-replication gap repair”) can be accomplished by translesion DNA synthesis, involving specialized translesion polymerases (reviewed in [1]), by template-switching to enlist an undamaged sister-strand as template [2, 3] or by homologous recombination (reviewed in [4]). Recruitment of DNA processing enzymes including nucleases, helicases and topoisomerases to a persistent gap, signaled by the presence of single-strand DNA binding protein (SSB), may also aid repair [5]. If left unrepaired, single-strand gaps are converted to potentially lethal double-strand breaks (DSBs) by endonucleolytic cleavage or by convergence of a new replication fork into the incompletely replicated region.

To study the process of ssDNA gap repair in *E*. *coli*, we have employed the nucleoside analog 3’ azidothymidine (AZT) [6], which acts as a chain terminator when incorporated into DNA by DNA polymerases. AZT arrests replication and causes single-strand gaps to accumulate in vivo, as evident by the cellular accumulation of foci of SSB protein. In addition, AZT promotes induction of the SOS DNA damage response, dependent on the RecFOR mediator proteins that promote RecA recombinase binding to single-strand gaps in DNA [6]. In *E*. *coli*, the genetic requirement for RecFOR is diagnostic for the formation of ssDNA gaps in adjoining duplex DNA and is distinct from single-strand DNA caused by resection of DSBs, which require RecBCD for processing and RecA loading (reviewed in [4]). *E*. *coli* cells can tolerate a certain level of AZT monophosphate incorporation, which appears to be excised from the 3’ nascent strain by Exonuclease III [6], a 3’ to 5’ exonuclease acting on duplex DNA from a nick or gap [7]. AZT also appears to elicit recombination via the RecAFOR pathway and to produce DSBs at some level, which are repaired via the alternative RecABCD recombination pathway [6].

To identify new functions that aid in AZT tolerance, we performed a genetic screen for expression-dependent suppression of AZT-sensitive phenotypes of several mutant strains. A plasmid library representing *tac*-promoter expressed *E*. *coli* open reading frames (ORFs) [8] was screened in pools for the ability to increase plating efficiency in the presence of normally-toxic levels of AZT. Two ORFs were recovered repeatedly: one encoding the putative Fe-S helicase, *yoaA*, and the other encoding an accessory protein of the replisome clamp loader (χ), *holC*. Interestingly, these proteins have been reported to interact physically by pulldowns of affinity-tagged bait proteins, followed by mass spectrometric analysis of interacting peptides [9]. We confirm this interaction in this study. The association of a DNA helicase with a replisome component provides a potential way to target a repair factor to a stalled replication fork.

In *E*. *coli*, the bulk of DNA replication is catalyzed by the DNA polymerase III holoenzyme, which participates in a plethora of protein interactions that regulate its activity and processivity (reviewed in [10]). The DNA polymerase III core complex (α, *dnaE*; θ, *holE*; and ε, *dnaQ*) is tethered to its template strand by the processivity clamp, β (encoded by *dnaN* gene). Loading and unloading of the clamp is facilitated by a clamp-loader complex, consisting of three subunits encoded by *dnaX* (τ and/or γ), and one each encoded by *holA* (δ) and *holB* (δ’). This clamp loader complex binds through τ to both the fork helicase protein, DnaB, and to the α subunit of the core DNA polymerase III complex. Two additional proteins, χ (encoded by *holC*) and ψ (encoded by *holD*), act in a dimeric complex as accessory proteins to the clamp loader [11]. Completing the cycle of interactions, ψ binds to γ or τ of the clamp loader [12] and χ binds to single-strand DNA binding protein, SSB, [13], that coats ssDNA revealed as fork unwinding proceeds.

The function of the accessory clamp loader proteins, χ and ψ, has been somewhat unclear since the core complex ([τ/γ]_3_δδ’) is sufficient to load and unload β clamps. However, a number of functions have been suggested by biochemical studies. χψ may aid assembly of the core clamp loader complex by increasing the affinity of τ/γ with δ and δ’ [14]. χ assists DNA polymerization on SSB-coated substrates [15] and promotes 5’ strand displacement [16]; the SSB-χ interaction may also aid the stability of Polymerase III on its template. χ promotes the handoff of primers from primase to PolIII [17]. ψ may also enhance clamp-loader activity and enforce an order to clamp assembly [18]. In *E*. *coli*, χ is nonessential (although mutants are quite sick and accumulate suppressors), and ψ’s essentiality can be suppressed by mutations that prevent the induction of the SOS response or by loss of translesion polymerases and the cell division inhibitor controlled by the SOS response [19]. The non-essentiality of χ and ψ are consistent with the hypothesis that χ and ψ are not obligatory for replication but replication in their absence causes ssDNA gaps to accumulate in the fork. This could result from defects in replication and/or the inability to elicit repair of gaps. Single molecule fluorescence microscopy of labeled replisome components suggests that χψ complexes are in excess of core polymerase complexes [20]. Therefore, χψ could be recruited independently through the SSB-χ interaction and provide a stand-alone, polymerase-independent function.

Our genetic studies reported here implicate the previously uncharacterized YoaA protein in the repair of replication forks and AZT tolerance. YoaA is related in protein sequence to a known *E*. *coli* Fe-S helicase of the Rad3/XPD family [21], DinG [22–24]. In addition, we show that a network of interactions is required for AZT tolerance: YoaA with χ, χ with SSB and ψ with χ. Our work suggests that persistent gaps in DNA accumulate SSB, which recruits YoaA. We propose that YoaA’s unwinding activity permits the proofreading exonuclease of Pol III to remove AZT from the nascent strand. Additionally, our work illustrates a new strategy by which replisome interactions enlist proteins directly to facilitate gap repair.

## Results

### Genetic suppressor screen and AZT-sensitivity phenotypes

To identify new repair factors, we performed a genetic screen for expression-dependent suppressors of the AZT sensitivity (AZT^s^) of various mutants defective in repair functions or their regulatory pathways. A pNTR- mobile plasmid library expressing each *E*. *coli* ORF (under the *tac* promoter) was introduced by conjugation into various AZT^s^ strains and those isolates that conferred increased tolerance to AZT were selected. We screened for suppressors of the following genetic mutants with different defects in repair: *xthA* (AZT excision), *recB* (DSB repair), *lexA3* (SOS regulatory response), *relA* (stringent regulatory response), *rpoS* (general stress response), *parE*-ts (Topoisomerase IV). Two ORFs were found repeatedly as suppressors in multiple screens: *yoaA*, an ORF of unknown function predicted to encode an Fe-S helicase, and *holC*, the χ subunit of the replication clamp loader complex. Both ORFs were isolated as suppressors of *xthA* and *parE*-ts; *holC* was isolated as a suppressor of *recB* and *yoaA* as a suppressor of *relA*, *rpoS* and *lexA3*. (Please note that the suppressor screen was not performed to saturation, so the failure to isolate an ORF from any particular strain does not mean it is not a suppressor.) Our interest in these suppressors was piqued by the fact that χ is a component of the replisome and has a reported physical interaction with the *yoaA*-encoded protein (b1808), documented in a high-throughput study of *E*. *coli* protein interactions [9].

**Fig 1:**
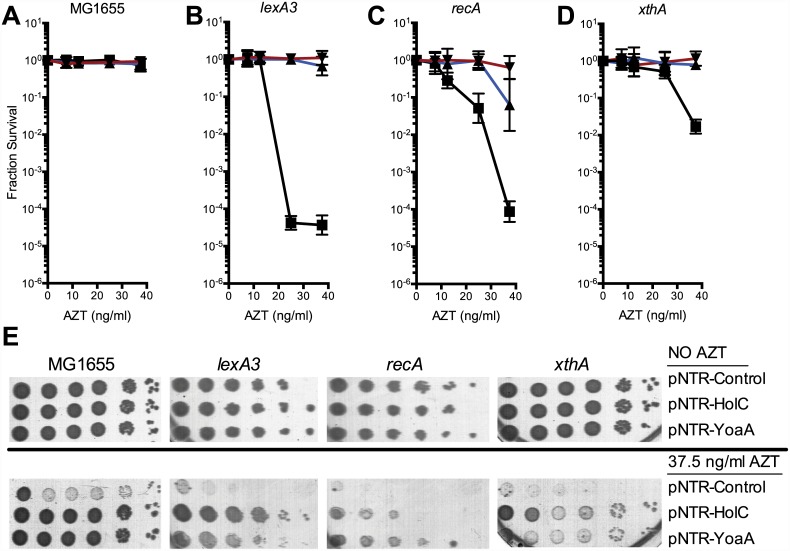
Survival of *lexA3*, *recA* and *xthA* mutants to AZT. (A) Fractional survival of MG1655 (wild-type) carrying the following plasmids: pNTR-Control, (closed square, black line); pNTR-HolC, (closed up triangle, blue line); or pNTR-YoaA, (closed down triangle, red line). (B) Fractional survival of *lexA3* carrying the following plasmids: pNTR-Control, (closed square, black line); pNTR-HolC, (closed up triangle, blue line); or pNTR-YoaA, (closed down triangle, red line). (C) Fractional survival of *recA* mutants carrying the following plasmids: pNTR-Control, (closed square, black line); pNTR-HolC, (closed up triangle, blue line); or pNTR-YoaA, (closed down triangle, red line). (D) Fractional survival of *xthA* carrying the following plasmids: pNTR-Control, (closed square, black line); pNTR-HolC, (closed up triangle, blue line); or pNTR-YoaA, (closed down triangle, red line). (E) Survival to AZT of MG1655 (wild type), *lexA3*, *recA*, or *xthA* carrying the following plasmids: pNTR-Control, pNTR-HolC, or pNTR-YoaA. Ten-fold serial dilutions of the indicated strains were plated on LB medium with or without AZT. Data represent n = 6 to n = 8 independent single colony isolates, shown as the average and standard deviation of log_10_-transformed fractional survivals. Representative images of plates are shown.

The ability of *holC* and *yoaA* to suppress the extreme AZT-sensitivity of several strains was determined by reintroduction of the mobile plasmids by DNA transformation. Plasmid-borne expression of either *holC* or *yoaA* dramatically increased tolerance to chronic AZT exposure (Fig 1) in a number of genetic backgrounds, including *lexA3* (defective in the SOS response), *recA* (defective in the SOS response and homologous recombination) and *xthA* (defective in exonuclease III). Expression of *holC* or *yoaA* improved AZT tolerance even in wild-type strains, as judged by colony size on AZT-containing medium (Fig 1), although *holC* or *yoaA* expression did not alter plating efficiency (i. e. the number of colonies that form). We hypothesize that *holC* and *yoaA* overexpression reduce the cellular burden of AZT by assisting in the removal of AZT-monophosphate from DNA. Expression of *holD*, encoding ψ, a partner to χ in the accessory clamp loader complex, or *holA* or *holB*, the δ and δ’ components of the clamp loader, did not alter AZT tolerance in the wild-type background (see S1 Fig). Expression of *dinG*, a DNA helicase and paralog of *yoaA*, was toxic and therefore could not be tested for suppressor activity (see S1 Fig).

**Fig 2:**
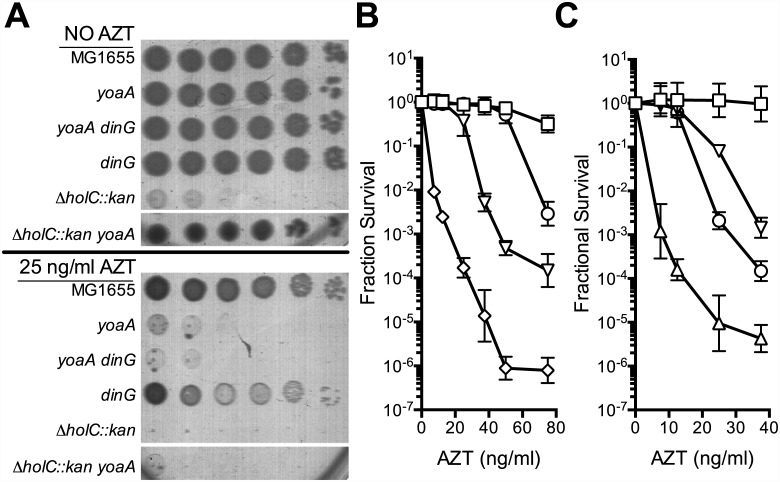
Survival of *holC*, *yoaA* and *dinG* mutants to AZT. (A) Plating of MG1655 (wild-type), *yoaA*, *yoaA dinG*, *dinG*, *ΔholC*::*kan*, or *ΔholC*::*kan yoaA* on LB medium with or without AZT. Ten-fold serial dilutions are shown from cultures grown and plated in parallel. Dilutions of other strains not relevant to this analysis were cropped out of these images. (B) Fractional survival of MG1655 (wild-type, open square), *yoaA* (open down triangle), *dinG* (open circle), or *yoaA dinG* (open diamond) mutant strains. (C) Fractional survival of MG1655 (wild-type, open square), *yoaA* (open down triangle), *holC* (open up triangle), or *yoaA holC* (open circle) mutant strains. Ten-fold serial dilutions of the indicated strains were plated on LB medium with or without AZT. Data represent n = 4 to n = 8 independent single colony isolates, shown as the average and standard deviation of log_10_-transformed fractional survivals.

**Fig 3:**
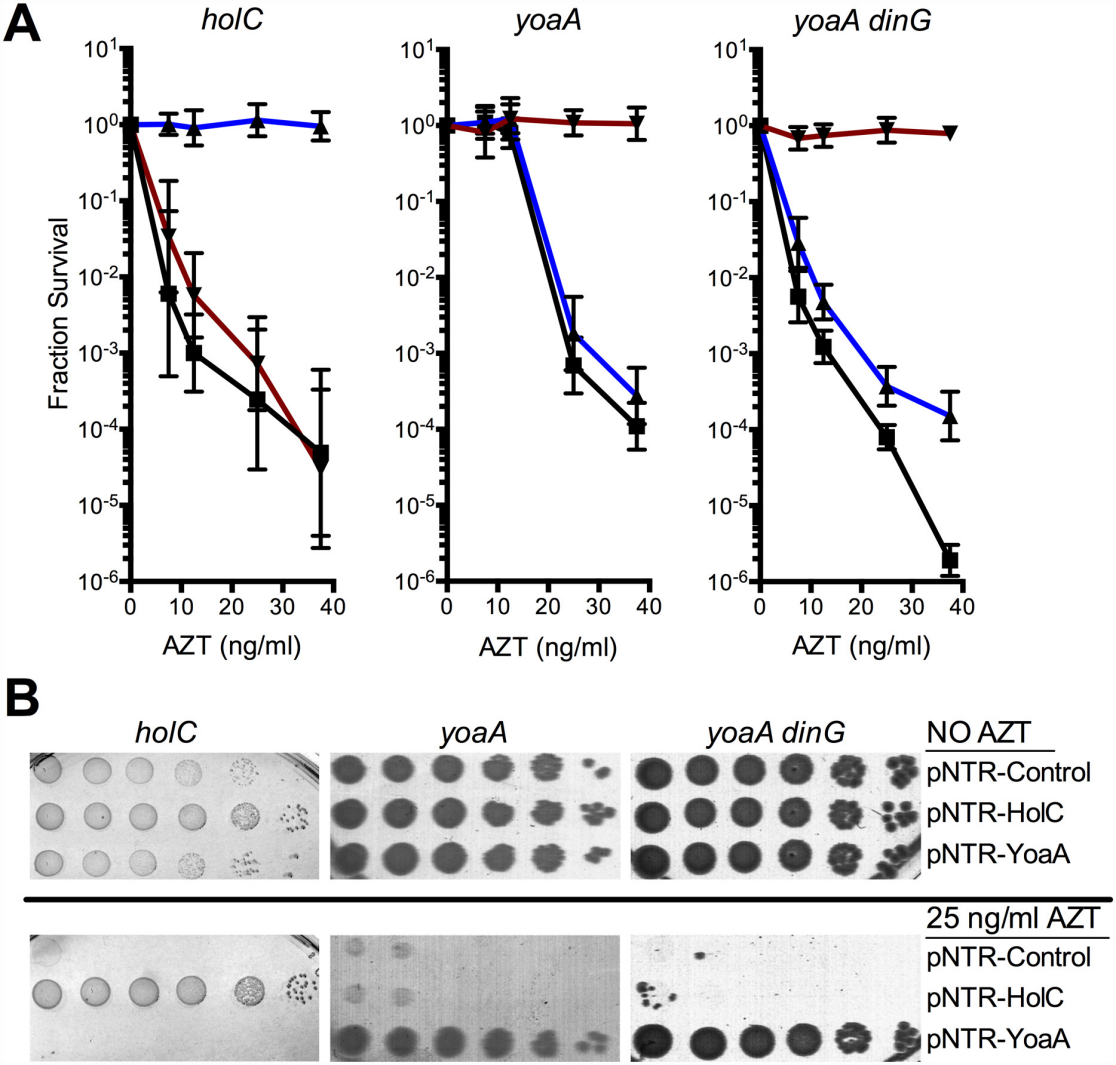
Survival of *holC*, *yoaA* and *yoaA dinG* mutants to AZT. (A) Fractional survival of *holCΔ*::*kan*, *yoaA*, or *yoaA dinG* mutants carrying the following plasmids: pNTR-Control, (closed square, black line); pNTR-HolC, (closed up triangle, blue line); or pNTR-YoaA, (closed down triangle, red line). (B) Survival of *holCΔ*::*kan yoaA*, or *yoaA dinG* mutants carrying the following plasmids: pNTR-Control, pNTR-HolC, or pNTR-YoaA. Ten-fold serial dilutions of the indicated strains were plated on LB medium with or without AZT. Data represent n = 6 to n = 8 independent single colony isolates, shown as the average and standard deviation of log_10_-transformed fractional survivals. Representative images of plates are shown.

Knockout mutants of *yoaA* and its paralog *dinG* were tested for effects on tolerance of AZT chronic exposure. Mutants in *yoaA* were sensitive to AZT, whereas mutants in *dinG* were sensitive only to very high concentrations of AZT. The double *yoaA dinG* mutant exhibited strong synergistic sensitivity to AZT (Fig 2). Sensitivity of both *yoaA* and *yoaA dinG* strains could be complemented by pNTR plasmid-expressed *yoaA* (Fig 3). These data indicate that YoaA plays a key role in AZT tolerance in wild-type *E*. *coli* cells, with DinG providing a partial backup function.

### Genetic and physical interaction of YoaA and χ

Because of the reported interaction between the YoaA and χ proteins, we tested whether *yoaA* suppression required *holC* and whether *holC* required *yoaA* (Fig 3). An *E*.*coli* strain with a deletion mutation in *yoaA* was strongly sensitive to AZT, with plating efficiency several orders of magnitude below wild-type strains (Fig 2). This phenotype was fully complemented by plasmid-expressed *yoaA* but *holC* expression did not suppress the phenotype (Fig 3). Likewise, a *holC* mutant poorly tolerates AZT exposure (Fig 2) and is complemented by plasmid-expressed *holC* but not suppressed by *yoaA* (Fig 3). This stands in contrast to other AZT-sensitive strains that were strongly suppressed by both *yoaA* and *holC* expression (Fig 1). The codependence of *holC* and *yoaA* suppression indicates that a YoaA/χ complex mediates tolerance to AZT and that the formation or effectiveness of this complex is enhanced by increased expression of either the YoaA or χ component. Interestingly, we note that the *holC* mutant, as assayed by plating efficiency, was more sensitive to AZT than the *yoaA* mutant; in fact, this increased sensitivity was ameliorated by loss of *yoaA* (Fig 2)—that is, functional YoaA leads to AZT-sensitivity in *holC* mutant strains. In addition, *yoaA* mutations appear to suppress the poor growth phenotype of the *holC* strain. Introduction of a YoaA-expressing plasmid into this *yoaA holC* strain re-sensitized it to AZT (S2 Table). These data indicate that YoaA function is, in some way, deleterious in the absence of χ, but not when χ is present.

**Fig 4:**
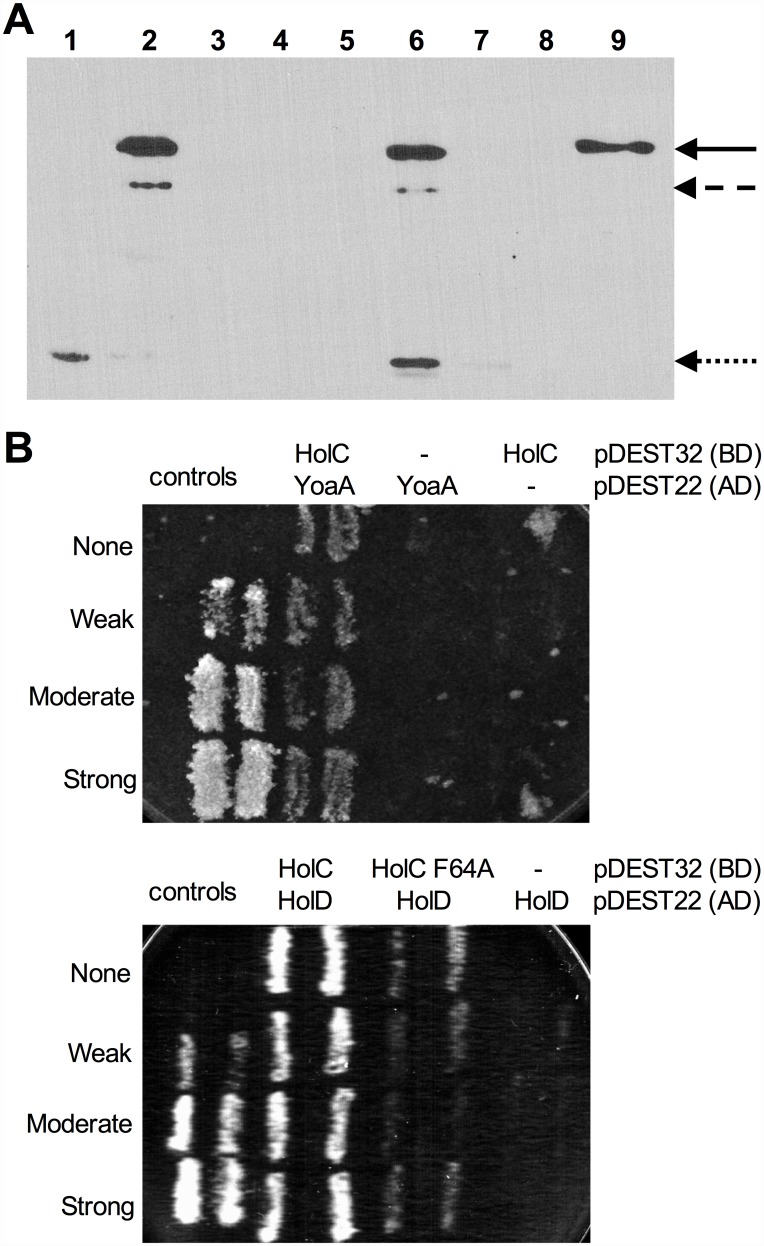
Confirmation of YoaA and HolC interaction. (A) Pull-down assays for interaction of YoaA-Biotin Binding Domain (BBD) to His_6_-HolC to Ni-NTA agarose resin. Samples were analyzed by Western blotting with NeutrAvidin Protein Horseradish Peroxidase Conjugated. (Lane 1) Crude lysate His_6_-HolC alone. (Lane 2) Crude lysate of YoaA-BBD alone. (Lane 3) Ni-NTA agarose resin alone. (Lane 4) Crude lysate His_6_-HolC mixed with Ni-NTA agarose resin with no crude lysate of YoaA-BBD added. (Lane 5) Crude lysate His_6_-HolC mixed with Ni-NTA agarose resin with no crude lysate of YoaA-BBD added washed. (Lane 6) Crude lysate His_6_-HolC mixed with Ni-NTA agarose resin with crude lysate of YoaA-BBD added. (Lane 7) Supernatant of wash of crude lysate His_6_-HolC mixed with Ni-NTA agarose resin with crude lysate of YoaA-BBD added. (Lane 8) Precision Plus Protein Standards. (Lane 9) Pull-down of crude lysate His_6_-HolC mixed with Ni-NTA agarose resin with crude lysate of YoaA-BBD added and washed. The solid arrow indicates full-length YoaA fused to BBD, at 77.4 kDa. The dashed arrow indicates a truncated form of YoaA, and the dotted arrow likely indicates BirA, biotin ligase. (B) YoaA/HolC and HolC/HolD interactions detected in a yeast two-hybrid system. *yoaA* ORF, *holC* ORF, mutant allele *holC F64A* ORF, and *holD* ORF were cloned into both pDEST32 (Gal4 DNA binding domain) and pDEST22 (Gal4 activation domain). The resulting plasmids were co-transformed with each other or with empty vector controls for pDEST32 and pDEST22 into the yeast strain *MaV203*. Control *MaV203* strains A (no interaction) containing pPC97 (no insert) + pPC86 (no insert); B (weak interaction) containing pPC97-RB + pPC86-E2F1; C (moderate interaction) containing pPC97-CH2^S^-dDP + pPC86-dE2F, E (strong interaction) containing pCL1 (encoding full-length Gal4) + pPC86. Qualitative yeast two-hybrid assay, *MaV203* control strains as well as strains containing plasmids encoding YoaA and HolC, HolC and HolD, or HolC F64A and HolD as described above, were plated on SC -Leu -Trp -His + 25mM 3-AT. Multiple streaks represent multiple strain isolates. Growth used Synthetic Complete (SC) -His -Leu -Trp medium, with plates containing 0.2% FOA, His 3-Amino-1,2,4-triazole (3-AT) as indicated.

A previous study employed mass spectrometry to identify *E*. *coli* proteins that interacted with 1000 TAP-tagged essential or conserved proteins [9]. This included *holC*-TAP, which copurified with a number of other proteins in the replisome and with YoaA (designated as “b1808” in that study). This study did not detect YoaA as an interactor with any other member of the clamp loader complex (including ψ, *holD*; γ/τ *dnaX*; δ, *holA*; or δ’, *holB*), suggesting that the χ:YoaA interaction might be direct. We sought to confirm this interaction by two means: by protein pulldown assays and by yeast two-hybrid analysis. We expressed a His_6_-tagged χ (HolC) protein (pCA24N-*holC*; [25]) in *E*. *coli* AG1 and immobilized the protein on a Ni-NTA resin. Extracts of *E*. *coli* BL21/DE3 expressing a biotin-binding domain (BBD)-YoaA fusion protein were then applied to the His_6_-HolC bound resin. Protein from input, wash and bound fractions were resolved by SDS-PAGE and subjected to Western blot analysis with Neutravidin-conjugated horseradish peroxidase to detect BBD-YoaA (Fig 4A). BBD-YoaA was indeed detected in the bound fraction (Fig 4A, lane 9), indicating a physical interaction, albeit possibly indirect, between χ and YoaA. A reciprocal pulldown experiment, using streptavidin-agarose resin to bind BBD-YoaA (bait) and a penta-His antibody to probe Western blots for His_6_-HolC (prey), likewise detected interacting His_6_-HolC. (S3 Fig). To detect a potential direct interaction, both *holC* and *yoaA* were subjected to yeast two-hybrid analysis (Fig 4B). An interaction between YoaA and HolC relative to controls was confirmed by the His phenotype of the strain. Because no other *E*. *coli* protein was expressed in the yeast assay strain, this latter result supports a direct physical interaction between χ and the YoaA protein.

### Mutational analysis of YoaA and χ suppression of AZT-sensitivity

**Fig 5:**
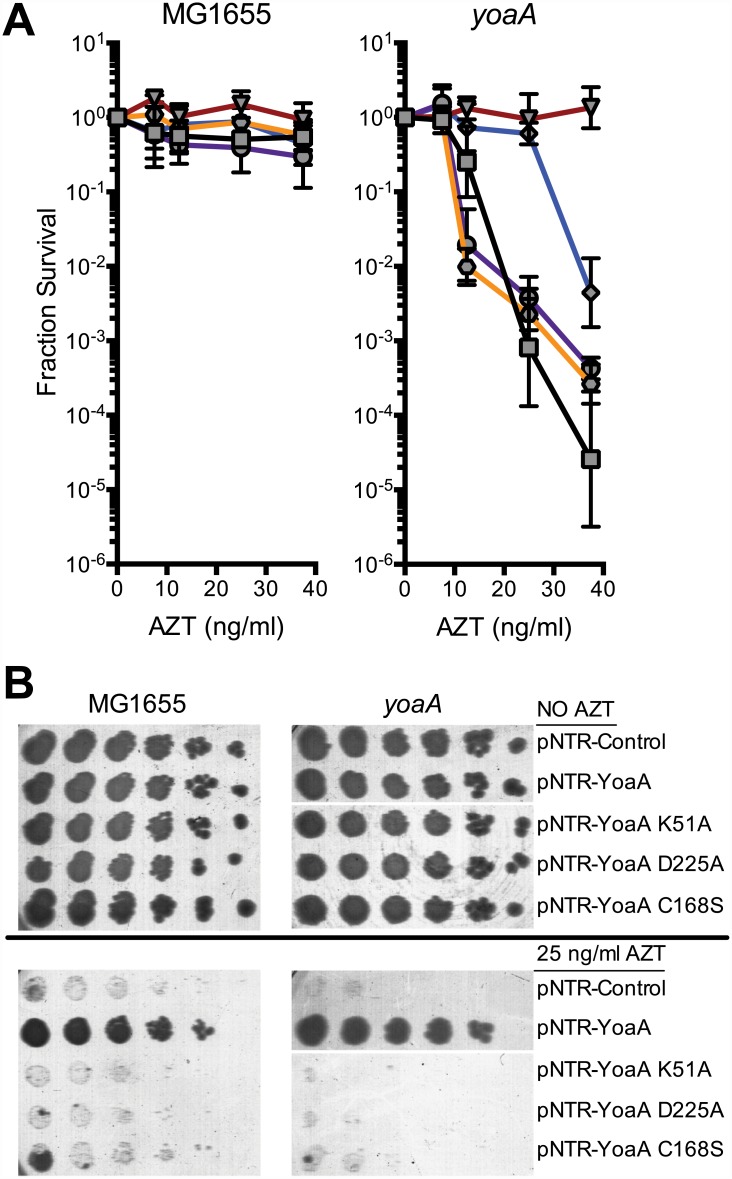
Survival to AZT of wild type and *yoaA* mutants expressing mutant YoaA on mobile plasmids. (A) Fractional survival of MG1655 (wild-type) or *yoaA* carrying the following plasmids: pNTR-Control, (square, black line); pNTR-YoaA, (down triangle, red line); pNTR-YoaA K51A, (hexagon, orange line); pNTR-YoaA D225A, (circle, purple line); or pNTR-YoaA C168S, (diamond, blue line). (B) Survival of MG1655 (wild-type) or *yoaA* carrying the following plasmids: pNTR-Control, pNTR-YoaA, pNTR-YoaA K51A, pNTR-YoaA D225A, or pNTR-YoaA C168S. Ten-fold serial dilutions of the indicated strains were plated on LB medium with or without AZT. Data represent n = 4 independent single colony isolates, shown as the average and standard deviation of log_10_-transformed fractional survivals. Representative images of plates are shown. Rows were from the same batch of plates in an assay done on the same day, rows of other strains not relevant to this figure were cropped out of these images.

Based on its amino acid sequence and similarity to DinG, YoaA is a predicted to possess 5’ to 3’ DNA helicase activity and a Fe-S cluster (See S2 Fig). We mutated the pNTR-plasmid borne *yoaA* gene within several motifs including the Walker A box (K51A), Walker B/DEAH box (D225A) and putative Fe-S coordination site (C168A), all of which are predicted to affect helicase function. These plasmid alleles, and the wild-type *yoaA* control plasmid, were introduced into wild-type and *yoaA* mutant strains. We assayed these strains for AZT tolerance, with expression of the plasmid allele induced by IPTG (Fig 5). All three mutant alleles fail to complement the AZT sensitivity conferred by *yoaA*. In wild-type strains, although plating efficiency is not affected, the size of colonies formed on AZT medium was dramatically reduced by the three mutant alleles of *yoaA*, in comparison to *yoaA* ^+^. These data support the notion that YoaA possesses ATPase activity essential to its genetic function (consistent with a helicase activity) and is likely also a Fe-S cluster containing protein, as is DinG.

**Fig 6:**
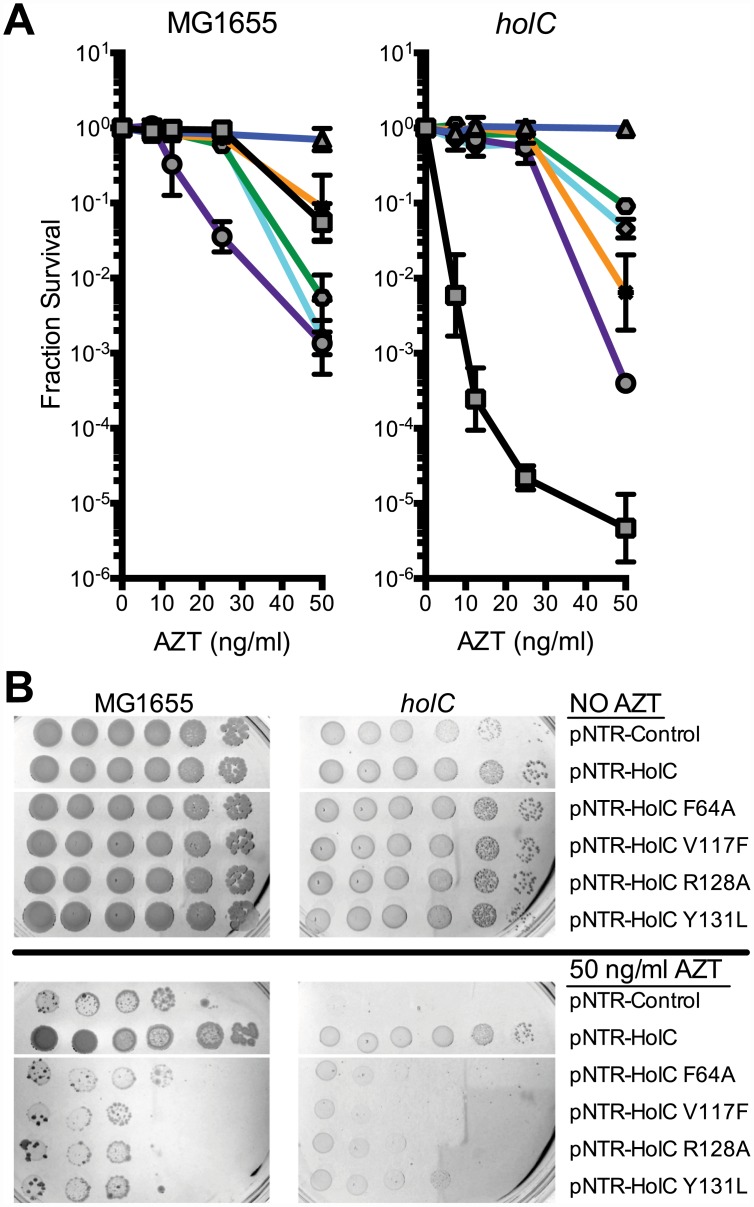
Survival to AZT of wild type and *holC* mutants expressing mutant HolC on mobile plasmids. (A) Fractional survival of MG1655 (wild-type) or *holCΔ*::*kan* mutant strains carrying the following plasmids: pNTR-Control, (square, black line); pNTR-HolC, (up triangle, blue line); pNTR-HolC F64A, (star, orange line); pNTR-HolC Y131L, (hexagon, green line); pNTR-HolC V117F, (circle, purple line); or pNTR-HolC R128A, (diamond, light blue line). (B) Survival of MG1655 (wild-type) or *holC* mutant strains carrying the following plasmids: pNTR-Control, pNTR-HolC, pNTR-HolC F64A, pNTR-HolC V117F, pNTR-HolC R128A, pNTR-HolC Y131L. Ten-fold serial dilutions of the indicated strains were plated on LB medium with or without AZT. Data represent n = 3 independent single colony isolates, shown as the average and standard deviation of log_10_-transformed fractional survivals. Representative images of plates are shown.

The χ protein participates in two additional protein interactions: one with its partner ψ (HolD) and with SSB. We sought to determine whether these interactions were necessary for suppression of AZT sensitivity by *holC* expression. A previous study [26] identified χ residues essential for SSB interaction, including V117, R128 and Y131 (Fig 6A and 6B). We introduced the V117F, R128A and Y131L mutations demonstrated to abolish SSB interaction [26] into the pNTR-*holC* construct and assayed their ability, relative to *holC* ^+^, to suppress AZT-sensitivity of wild-type or *holC*Δ strains. The SSB-binding-defective mutants had reduced ability to suppress the AZT-sensitivity of *holC*, as illustrated by both plating efficiency and colony size on AZT-containing media. These SSB-interaction mutants, when expressed in *holC* mutant strains did show increase in plating efficiency relative to the control plasmid (Fig 6A); however colonies formed on AZT-medium were small and slow-growing (Fig 6B). These mutants, particularly the V117F allele, also showed dominant reduction of AZT tolerance in wild-type strains, apparent by both plating efficiency (Fig 6A) and colony size (Fig 6B). Assuming that these substitutions do not affect protein folding or expression levels (which is consistent with their genetic dominance), this result confirms that the ability of χ to interact with SSB is critical to the mechanism that promotes AZT tolerance. Residues in χ that are required for its interaction with ψ have not been reported. However, the crystal structure of the χψ dimeric complex [11] suggests that χ-F64 is a good candidate since it appears buried in a hydrophobic pocket at the χ:ψ interface. We mutated pNTR-*holC* to carry a F64A mutation and showed that this mutation abolishes AZT tolerance promoted by pNTR-*holC* expression in both wildtype and *holC*Δ mutant strain backgrounds (Fig 6). To confirm that this mutation indeed affects the interaction between χ and ψ, we subjected *holC* and *holC*-F64A to yeast two-hybrid analysis with *holD* (Fig 4B). HolC and HolD exhibit an interaction comparable to “strong interaction” controls, with HolC-F64A significantly reducing the interaction. Therefore, suppression of AZT sensitivity by HolC expression appears to require an intact χψ complex.

### Genetic interaction with exonucleases

**Fig 7:**
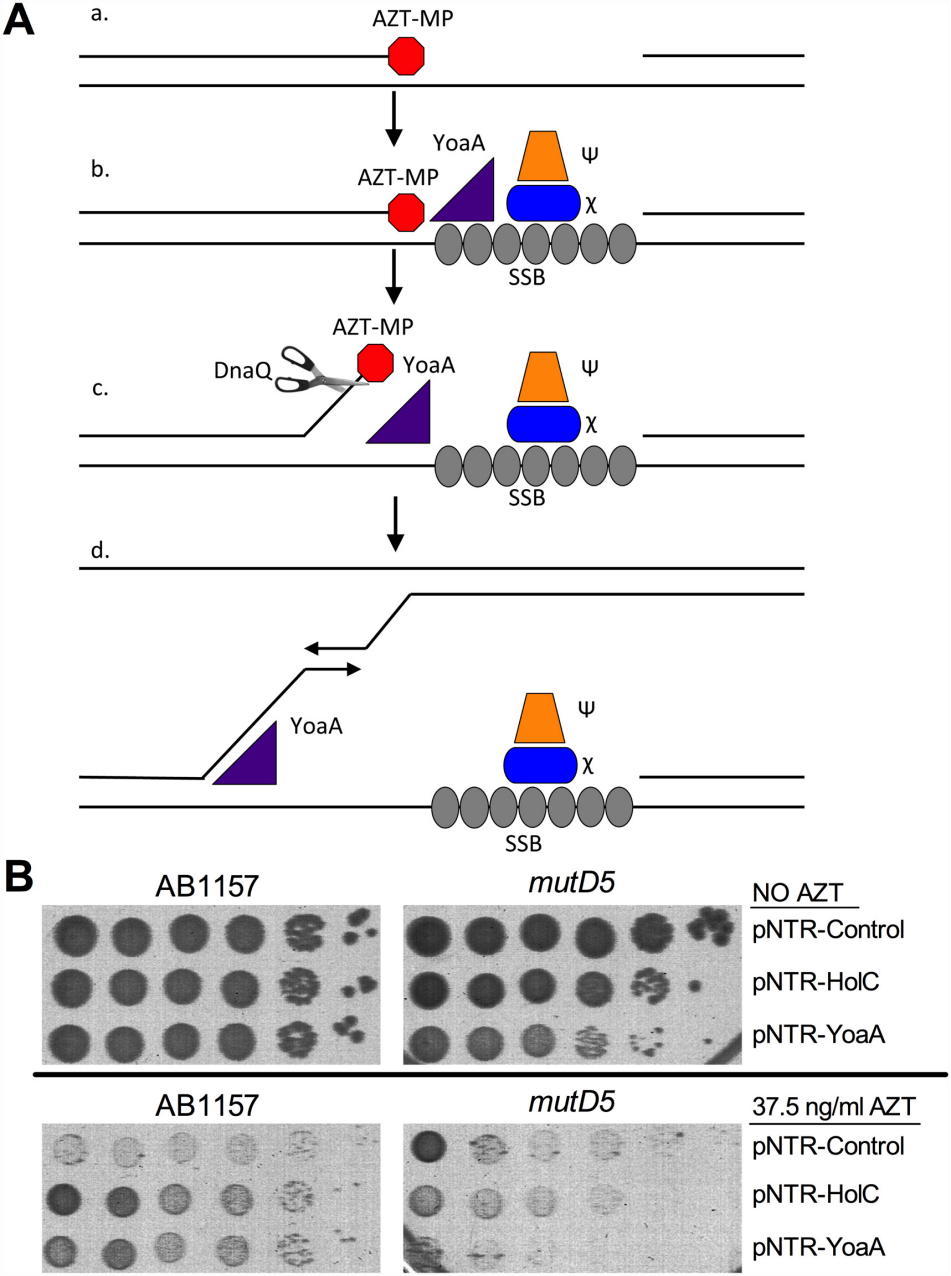
Model for the role of YoaA, x (HolC) and ε (DnaQ) in AZT tolerance. (A) (a) AZT-incorporation by DNA Polymerase III during replication blocks 3’ extension of nascent strand, producing a ssDNA gap. Although not shown, this gap on the lagging strand would be pre-bound by SSB (b) SSB at the gap recruits χ and ψ to the site of damage. YoaA is recruited by χ to the site of damage where it then (c) unwinds the nascent strand to facilitate removal of AZT by the proofreading exonuclease ε (DnaQ) subunit of the DNA Polymerase III holoenzyme. (d) YoaA may also stimulate template switching through its unwinding of the nascent strand. Although not shown, other DNA polymerase III holoenzyme components may be recruited through clamp-loader interactions. (B) Survival of AB1157 (wild-type) or *mutD5* (mutant allele of DnaQ), carrying the following plasmids: pNTR-Control, pNTR-HolC or pNTR-YoaA. Ten-fold serial dilutions of the indicated strains were plated on LB medium with or without AZT. Representative images of plates are shown.

In several bacterial groups including Gram-positives, the ortholog to YoaA/DinG helicase is fused to an N-terminal exonuclease domain from the DnaQ/ε 3’ exonuclease family [27]. Our prior genetic study of AZT tolerance in *E*. *coli* suggests that Exonuclease III, a dsDNA exonuclease that degrades a 3’ strand from nicks or gaps in DNA, is likely the enzyme that removes AZT monophosphate from the DNA chain [6]. Since YoaA and χ expression strongly suppressed AZT sensitivity of *xthA* mutant strains (Fig 1), we considered the possibility that χ recruitment of YoaA helicase to SSB-bound gaps might permit an alternative 3’ ssDNA exonuclease to remove AZT by unwinding the 3’ nascent strand from its template (Fig 7). In Gram-positive and other groups of bacteria (see Discussion below), this exonuclease is conveniently fused to the helicase polypeptide. This hypothesis predicts that loss of a 3’ exonuclease would weaken the ability of YoaA or HolC expression to promote AZT tolerance. *E*. *coli* possesses 8 members of the DnaQ exonuclease family including the proofreading activities for DNA polymerases I (*polA*), II (*polB*) and III (*dnaQ*), exonuclease I (*xonA*) and exonuclease X (*exoX*).

DnaQ itself emerged as a strong candidate for the YoaA-associated 3’ exonuclease. Suppression of AZT sensitivity by YoaA or HolC was strongly reduced by the *mutD5* allele of the *dnaQ* gene (Fig 7). (The *mutD5* allele of *dnaQ* is a T15I mutation adjacent to the catalytic glutamate in the Exo I motif of the protein [28]. This allele reduces exonuclease activity without affecting its association with the polymerase subunit α [29]; mutation of the catalytic glutamate is lethal [28].) Moreover, YoaA expression became somewhat toxic in *mutD5* strains, evident by lower plating efficiency and smaller colonies of *mutD5* strains, both on LB and LB AZT media. We explain the latter result by hypothesizing that single-strand DNA displaced by YoaA is deleterious when it fails to be degraded by DnaQ (see Discussion below).

## Discussion

This work provides new evidence that the accessory proteins to the clamp loader complex play a role in coordination of DNA repair. Through a chain of protein interactions, the accessory clamp loader proteins χ and ψ may facilitate the binding of multiple repair and replication factors. Persistent unfilled gaps are likely to be bound by SSB, which recruits χψ. χ binds the YoaA helicase, which we hypothesize unwinds potentially damaged 3’ nascent ends such as those terminated by azidothymidine monophosphate. Our work suggests that YoaA permits ε, encoded by DnaQ, to degrade the 3’ nascent strand end. Whether this degradation occurs in the context of the fully assembled replisome, an αεθ Polymerase III core complex, or by a stand-alone ε subunit is not known. Through its ψ interaction with τ or γ, χψ recruits the clamp loader to the gap for ready assembly of β clamps at the site of the gap. These clamps may then bind repair factors such as MutLS, ligase, TLS polymerases or recruit DNA polymerase III to fill the gap. These interactions may aid rapid hand-off of repair intermediates, promoting maturation of a persistent gap into a repair substrate and then into a replicative complex. Association of YoaA with χ may also allow it to track with the fork, even in the absence of persistent SSB-bound gaps.

We define here a new potential DNA repair function, encoded by the previously uncharacterized gene *yoaA*, that aids in tolerance of the chain-terminating nucleoside, azidothymidine. We confirm by protein pull-down and yeast two-hybrid analysis that YoaA binds χ of the accessory clamp loader complex. Genetic analysis demonstrates that YoaA’s putative ATPase activity is important for its in vivo activity. YoaA is likely to function in DNA repair as a 5’ to 3’, Fe-S helicase, similar to its paralog, DinG. *E*. *coli* YoaA and DinG share 29% identity (see alignment in S2 Fig) over almost their entire length, including the four cysteines in the DinG Fe-S coordination site, C120, C194, C199, C205 [24], and helicase motifs. The *dinG* gene was discovered as a DNA damage-inducible gene, regulated by the LexA repressor as part of the SOS response [29–31]. DinG is a member of a large group of helicases, including the human excision repair factor XPD, and helicases FANCJ/BACH-1, RTEL1 and ChlR1/2 [21, 31]. Mutations affecting these human helicases are associated with a variety of human DNA repair-deficient genetic diseases (reviewed in [27]). The translocation direction of *E*. *coli* DinG on ssDNA is 5’ to 3’ and its helicase activity is somewhat structure-specific, with highest activity in the unwinding of bifurcated, fork-like structures. Both RNA:DNA and DNA:DNA hybrid molecules can be unwound by DinG [23].

DNA helicases may help avoid the formation of replication gaps or facilitate their subsequent repair by several means. Helicases may unwind DNA structures, such as hairpins or G- quadruplex structures, which impede polymerization or fork progression. They may displace bound proteins, including RNA polymerase, unwind stable RNA:DNA hybrids (“R-loops”) or disassemble protein complexes formed during repair. Helicases may promote template-switching reactions that function in repair. Helicases also process branched DNA structures that are considered to be intermediates in homologous recombination such as D-loops and Holliday junctions and therefore function in recombinational DNA gap repair. Mycobacterial DinG has been reported to unwind G-quadruplex structures, in vitro [32]. DinG is one of several helicases, including Rep and UvrD, that are required in *E*. *coli* to overcome problems associated with DNA replication clashes with strong transcription [33].

YoaA likely differs from DinG in that YoaA is associated with the replisome, through its χ interaction, although it remains possible that the two helicases possess specialized binding affinities or activities. We observed expression-dependent suppression of AZT sensitivity by YoaA probably because of the increased likelihood of association with the χψ complex and timely recruitment to gaps when intracellular levels of YoaA are increased. An increase in intracellular χ might also make YoaA recruitment more efficient. In the study that identified the YoaA:χ interaction, DinG was not detected as a replisome-associated polypeptide [9]. DinG’s weak AZT-sensitive mutant phenotype and genetic synergy with YoaA suggest that it can play a similar role to YoaA in repair, albeit more inefficiently. This inefficiency may result from an intrinsic difference in the binding or helicase activities of the DinG and YoaA or from the fact that YoaA is more efficiently recruited to gaps through its χ interaction. The sites within YoaA that interact with χ will be of interest, but are currently unknown. Our pull-down assays lead us to suspect that the C-terminus will be important since what we infer is a C-terminally truncated BBD-YoaA form, detected in our bacterial cell extracts by Western blot analysis, did not appear competent to interact with χ. Our genetic analysis supports the notion that YoaA interacts with the χψ complex rather than with χ alone in the absence of ψ. The consequences of χ:YoaA binding on χψ ‘s other interactions, such as to the clamp loader or to SSB, will be important topics for future investigation. We may find, for example, that YoaA interactions with χψ are in competition with the DnaX clamp-loader interaction, or alternatively, they may be neutral or even enhance each other’s recruitment.

A number of observations reported here support the idea that YoaA, when liberated from association with other factors, can be toxic. Mutants lacking χ are very sensitive to AZT exposure, a phenotype partially suppressed by loss of YoaA. In the absence of the χ targeting factor, YoaA may be deleterious to repair and cell survival. Additionally, induction of YoaA expression is toxic to cells with defective DNA Polymerase III proofreading (*mutD5*), particularly after AZT exposure. This toxicity may be related to genetic instability, the DNA damage response and/or toxic reactions promoted by accumulation of single-strand DNA. This situation may be analogous to the cold-sensitivity of ssDNA exonuclease mutants (lacking RecJ, ExoI, VII and X), in which toxicity is dependent on UvrD helicase function [34]. The toxicity of ssDNA may result from constitutive induction of the SOS response [35] and/or by stimulation of low-homology recombination reactions [36] or template-switching [2] that lead to genetic mutations or chromosome rearrangements. We propose that the physical interactions that target YoaA to the replication fork and promote the handoff of repair intermediates to replication functions act to control the potential toxicity of strand unwinding.

Many diverse bacterial genomes encode a putative helicase related to DinG and YoaA of *E*. *coli*, leading to the hypothesis that it evolved early in the history of life and plays an important role in bacterial cell fitness. Although *E*. *coli* and other γ-Proteobacteria encode two paralogs, many bacteria possess only a single member of this group. The β-Proteobacteria encode a single YoaA ortholog, with over 95% identity to *E*. *coli* YoaA and only about 40% to *E*. *coli* DinG. The α and δ-Proteobacteria encode a single protein more distantly related to both proteins (about 40% identical to YoaA and 30% to DinG). In Gram-positive, Firmicutes bacteria (such as *Bacillus subtilis)* and in the Thermus-Deinococcus, green nonsulfur and Fusobacteria groups, the YoaA/DinG related helicase is fused to an N-terminal domain with homology to the DnaQ/ε (DEDDh) family of 3’ exonucleases. Although these proteins have been termed in various databases as DinG orthologs, protein sequence alignments support the notion that they are more strongly related to *E*. *coli*’s YoaA protein than its DinG protein. S2 Fig shows the pairwise BLAST alignment between the *B*. *subtilis* protein and *E*. *coli* YoaA, compared to the alignment of *E*. *coli (Eco)* YoaA and DinG, showing multiple regions that align to YoaA but not DinG. This homology is apparent in the gapped regions shared by *Bsu* ε and *Eco* YoaA, relative to *Eco* DinG, particularly in the region between helicase motifs II and III. Overall identity of *Bsu* ε with *Eco* YoaA or DinG is 29% and 25%, respectively, with 10% or 14% gaps. The *Bacillus* protein does not retain the conserved cysteine residues shared by *Eco* YoaA and DinG that are required to form the Fe-S cluster in this helicase family and so is unlikely to be an Fe-S protein.

Our previous work implicated the 3’ dsDNA exonuclease, Exonuclease III, as an enzyme that removes AZT monophosphate (AZT-MP) from DNA in *E*. *coli* [6]: exonuclease III mutants are highly sensitive to normally sublethal concentrations of AZT and overproduction of the enzyme improves tolerance in wild-type strains. In this study, we found that mutants in the proofreading activity of DNA polymerases I or III show little or no sensitivity to AZT. This may be because AZT-MP is poorly proofread or, alternatively, because loss of proofreading is efficiently backed up by exonuclease III-mediated removal. We have no information about how efficiently AZT-MP can be removed in vitro from DNA by bacterial DNA polymerases. However, in both yeast and humans, AZT-MP is poorly proofread by DNA polymerase gamma, a DNA polymerase that readily incorporates AZT [37,38]. The work reported here suggests that YoaA DNA helicase activity aids removal of AZT-MP from 3’ termini by DnaQ, the proofreading subunit of DNA Pol III. When Pol III is bound to the paired template and nascent strand, AZT-MP may be poorly accessible to the exonuclease active site of the DnaQ because of AZT’s bulky 3’ azido group and/or because it pairs well with its template adenine residue. YoaA helicase may promote the dissociation of Pol III and/or unwind the nascent strain from its template (as shown in Fig 7), allowing it or a second enzyme to gain access to AZT at the 3’ nascent strand terminus.

## Materials and Methods

### Bacterial strains and growth conditions

*E*. *coli K-12* strains isogenic to either the wild type MG1655 (F- *rph-1*) or wild type AB1157 background (S1 Text) were grown as previously described at 37° in Luria-Bertani (LB) medium, with 1.5% agar for plates [39]. With the exception of *lexA3*, *parE-ts* and *mutD5*, all mutant strains used in this study carried deletions of the indicated gene. LB medium was supplemented as necessary with antibiotics including ampicillin (Ap), chloramphenicol (Cm), kanamycin (Km), tetracycline (Tc), streptomycin (Sm) or gentamycin (Gm). Details of growth media and strain constructions are provided in the S1 Text.

### Mobile plasmid genetic screen

Details of the screen can be found in the S1 Text. Briefly, screens were performed with MG1655 isogenic strains carrying mutations that confer increased AZT sensitivity, including *xthA*, *recB*, *rpoS*, *lexA3*, *relA*, *parE*-ts (grown at 30°). The plasmid library of pNTR-based mobile plasmids carrying *E*. *coli* ORFs [8] was obtained from the National Bioresource Project at the National Institute of Genetics in Japan, which were introduced into tester strains in pools and screened for suppression of AZT sensitivity. These plasmids are based on a ColE1 replicon, with a copy number of approximately 30 per cell.

### Survival assays

Isolated mobile plasmids (S1 Table) were transformed into strains by electroporation [40]. For survival assays, strains were grown to OD_595_ 0.3–0.7, serial diluted in 56/2 buffer, and then plated on LB agar plates supplemented with azidothymidine (AZT) as indicated. Plates were incubated at 37° for 24–36 hours. Total colony forming units (CFU) counts were obtained, normalized to the NO AZT counts, and then log_10_-transformed to obtain Fractional Survival. Averages and standard deviations were calculated from the log_10_-transformed Fractional Survival data, and plotted as a function of AZT dosage. Strains containing mobile plasmids pNTR-Control, pNTR-HolC, or pNTR-YoaA were plated on growth medium supplemented with Ap, and expression was induced by the addition of 1 mM IPTG. Because of day-to-day variation of the potency of AZT, assays illustrated in the figure panels were conducted in parallel.

### Mutant alleles

Site-directed mutagenesis (Quikchange, Agilent Technologies) was used to construct mutant alleles of *holC* and *yoaA* on the pNTR mobile plasmids, as well as *holC* plasmid derived from pDONR221 by GATEWAY cloning (Life Technologies). Primers used to construct the site-directed mutant alleles are listed in S1 Table.

### Yeast two-hybrid analysis

We used a commercially available yeast two-hybrid system (ProQuest, Life Technologies) to detect interactions between YoaA and HolC and between HolC and HolD when C-terminally fused to Gal4 DNA binding and activation domains. Details of the assays are provided in the Supplement. Readouts for transcriptional activation include *URA3*, *HIS3* and *lacZ* expression in yeast strain MaV203. Controls include MaV203 “no interaction” strains containing pPC97 (no insert) + pPC86 (no insert); “weak interaction” strains containing pPC97-RB + pPC86-E2F1; “moderate interaction” strains containing pPC97-CH2^S^-dDP + pPC86-dE2F, and “strong interaction” strains containing pCL1 (encoding full-length GAL4) + pPC86. Primers used to construct GATEWAY cloned alleles of *yoaA*, *holC*, and *holD* are listed in S1 Table.

### Protein extracts, pull-down assays and Western blot analysis to display YoaA and HolC protein interaction binding

Plasmid pSTL385 is a pET104.1-DEST based plasmid (Life Technologies) comprised of a N-terminal fusion of the biotin binding-domain (BBD) to *yoaA*, expressed from the T7 promoter. BBD-YoaA was expressed from *E*. *coli* B strain BL21 (DE3) (genotype *fhuA2 lon ompT gal dcm hsdS* λ DE3), grown in LB + Ap medium, and induced by the addition 1 mM IPTG for 2 hours. Plasmid pSTL386 is a pCA24N-based plasmid comprised of a N-terminal His fusion to *holC* under the *lac* promoter [41], which was transformed and expressed in strain AG1 (*recA1 endA1 gyrA96 thi-1 hsdR17 supE44 relA1*). The strain was grown in LB + Cm medium, induced by 1 mM IPTG for 2 hours. Cells of both overexpressing strains were harvested by centrifugation, resuspended and stored in Tris-sucrose buffer (50 mM Tris-HCl pH 7.5 10% sucrose) at -70°. Crude cell extracts were prepared by lysozyme lysis as described previously [42]. Pulldown of His_6_-HolC was performed from crude cell extracts with Ni-NTA agarose beads (Qiagen), equilibrated in wash buffer (50 mM Na_2_HPO_4_/NaH_2_PO_4_ pH 8.0, 500 mM NaCl, 20 mM imidazole). Resin was mixed with an equal volume of the crude cell extracts from the His_6_-HolC expressing strain, allowed to incubate at 20° for 1 hour and was then washed five times with wash buffer. YoaA-BBD crude lysate was similarly mixed with the His_6_-HolC bound resin, incubated and washed. Samples were eluted by mixing the resin 1:1 in 2x Laemmli sample buffer (120 mM Tris-HCI, pH 6.8, 4% SDS, 40% (w/v) glycerol, 0.02% bromophenol blue). Protein samples were subject to PAGE in 15% polyacrylamide gels and transferred to PVDF membrane transfer using a Mini Trans-Blot Electrophoretic Transfer Cell (Bio-Rad) and the methods provided by the manufacturer. The Western blot analysis was performed using the QIAexpress detection kit and protocol (Qiagen) and using detection of BBD-YoaA with a 1:10,000 dilution of NeutrAvidin Protein Horseradish Peroxidase Conjugated antibody (Pierce).

## Supporting Information

S1 Text Supplemental materials and methods. Bacterial strain construction, growth and protein interaction methods.
(DOCX)

S1 Table Strains, plasmids and oligonucleotides used in this study. **Table S1A**. **Strains and plasmids. Table S1B. Oligonucleotides.** Sequences are presented 5’ to 3’.
(DOCX)

S2 Table Fractional survival of *holC* and *holC yoaA* strains expressing mobile plasmids on medium containing AZT. Relative plating efficiency on LB medium containing the indicated AZT concentrations relative to that on LB medium without AZT.
(DOCX)

S1 Fig Survival to AZT of wild type strains with mobile plasmids. Survival to AZT of MG1655 (wild type) carrying the following plasmids: pNTR-Control, pNTR-DinG, pNTR-YoaA, pNTR-HolC, pNTR-HolD, pNTR-HolA, pNTR-HolB, or pNTR-XthA.
(EPS)

S2 Fig BLAST amino acid sequence alignment between *Bacillus subtilis* ε and *E. coli* YoaA and between *E. coli* YoaA and DinG proteins. Above the alignment are indicated the conserved helicase motifs and below, with asterisks, the Fe-S coordination residues for DinG.
(TIF)

S3 Fig Investigation of YoaA and HolC interaction, reciprocal pulldown. Pull-down assays for interaction of His_6_ HolC to YoaA-Biotin Binding Domain (BBD) to streptavidin agarose resin. (Lane 1) YoaA-BBD crude lysate alone. (Lane L) Bio-Rad Dual Colored Protein Standards. (Lane 2) Crude lysate His_6_-HolC alone. (Lane 3 & Lane 4) Streptavidin agarose resin + YoaA-BBD lysate. (Lane 5 & Lane 6) Streptavidin agarose resin + YoaA-BBD lysate + His_6_-HolC lysate. (Lane 7 & Lane 8) Streptavidin agarose resin + YoaA-BBD lysate + His_6_-HolC, washed 5 times and eluted. The solid arrow indicates HolC fused to His_6_, at 21 kDa. Fainter and larger species in lanes 1, 2, 5, 6 represent crossreacting proteins to the pentaHis primary antibody that are removed in the wash step.
(EPS)
