# Supplementary material for: Connecting Replication and Repair: YoaA, a Helicase-Related Protein, Promotes Azidothymidine Tolerance through Association with Chi, an Accessory Clamp Loader Protein
Source: PLoS Genet. 2015 Nov 6;11(11):e1005651. doi: 10.1371/journal.pgen.1005651 (PMC4636137; doi:10.1371/journal.pgen.1005651)
Supplement: S1 Text — Bacterial strain construction, growth and protein interaction methods. (DOCX) [file pgen.1005651.s001.docx]

**SUPPORTING INFORMATION TEXT**

**Supplemental materials and methods**

**Bacterial strains and growth conditions:** *E. coli K-12* strains isogenic to either the wild type MG1655 (F- *rph-1*) or wild type AB1157 background are listed in supplemental Table S1. P1*vir*a transductions were performed as described [1] and selections employed are listed in Table S1A below. Antibiotics were used in the following concentrations: 100 μg/ml ampicillin (Ap), 15 μg/ml chloramphenicol (Cm), 60 μg/ml kanamycin (Km), 15 μg/ml tetracycline (Tc), 100 µg/ml streptomycin (Sm), 100 µg/ml gentamycin (Gm). Strains were constructed by P1 transductions using LCG medium (LB supplemented with 1% glucose, 2 mM calcium chloride, and 1% agar for plates. Plasmid mutant alleles were constructed by site-directed mutagenesis using Quikchange kits (Agilent Technologies). Knockout mutations were constructed as described [2] using oligonucleotides listed in Table S2 and plasmids pKD46 (to provide Red recombination functions), pKD3, pKD4 (PCR templates for *kan* and *cat*-linked disruptions) and pCP20 (encoding FRT recombinase to cure drug resistance markers flanked by FRT sites). Constructions were confirmed by PCR.

**Mobile plasmid genetic screen:** Initial screens were performed with MG1655 isogenic strains carrying mutations that confer increased AZT sensitivity, including *xthA* (STL10183), *recB* (STL7255), *rpoS* (STL7291), *lexA3* (STL12071), *relA* (STL8198), *parE*-ts (STL14019). The *parE*-ts sceen was conducted with 30° growth, whereas the remaining screens were performed at 37°. An ordered library of pNTR-based mobile plasmids carrying *E. coli* ORFs [3] was obtained (through the National Bioresource Project at the National Institute of Genetics in Japan) in strain background JA200 (F+ *thr-1 leuB6 trpEΔ5 recA lacY thi gal mtl ara xyl*). 48 mobile plasmid-bearing strains (pNTR-ORF clones) were pooled and each pool was placed in a single well of a 96-well deep-well microtiter dish and grown to saturation in LB medium, to allow all *E. coli* ORFs to be screened using a single 96-well plate. The pooled cultures were frozen in 15% glycerol at -70°C. Donor pool cultures in standard microtiter dishes were prepared by inoculation into LB from the frozen pooled stocks, the plate was then covered with a gas-permeable sterile membrane (Breathe-Easy, Sigma-Aldrich) and incubated overnight at 37°C. The plasmids were introduced by conjugation into the AZT-sensitive tester strains by mixing each 5 µl of the overnight donor strain pools with 100 µl overnight recipient culture in LB and incubating for 1 hr. At that time, using a 48-prong metal replicator device (Replica Plater R2383, Sigma-Aldrich) that delivers approximately 2 µl, cultures were diluted 10^-2^ and 10^-4^ in 56/2 buffer [4] in 96-well dishes. Each dilution and the undiluted mixture were plated using the 48-prong replicator on LB Ap Sm medium containing IPTG at 1 mM and AZT at predetermined concentrations selective for each strain, ranging from 12.5 to 100 ng/ml. (Ap was used to select for the mobile plasmid while Sm was used to select the recipient strains and counter-select the donors; IPTG induces the *E. coli* ORF expression) Colony isolates with AZT-resistant survivors exceeding the background frequency were recovered and streaked on LB Ap Sm medium to obtain individual colonies. Plasmid DNA was purified (Qiagen) and subjected to DNA sequence analysis (Genewiz) to identify the ORF. Backcrosses involving reintroduction of the plasmid by DNA electroporation transformation [5] into the recipient strains were performed to confirm suppression.

**Mutant alleles:** Primers used to construct the site-directed mutant alleles are listed in Table S1B below.

**Western Blot Analyses of reverse pulldown:** The reverse pulldown was performed with extracts obtained as described in the *Protein extracts*, *pull-down assays and Western blot analysis to display YoaA and HolC protein interaction binding* methods section. In this experiment, however, the YoaA-BBD crude lysates were incubated for one hour at 20°C with streptavidin agarose resin (Life Technologies) that had been equilibrated in wash buffer (50 mM Na_2_HPO_4_/NaH_2_PO_4_ pH 8.0, 500 mM NaCl, 20 mM imidazole). Resin-immobilized proteins were washed five times with wash buffer, and then incubated for one hour at 20°C with an equal volume of the crude cell extracts from the His_6_-HolC expressing strain. Following five washes with wash buffer, proteins bound to the YoaA-BBD/streptavidin-agarose resin complex were eluted in SDS-PAGE sample loading buffer by mixing the resin 1:1 in 2x Laemmli sample buffer (120 mM Tris-HCI, pH 6.8, 4% SDS, 40% (w/v) glycerol, 0.02% bromophenol blue). Samples were then loaded in individual wells on a handcast SDS-PAGE gel (6% acrylamide stack, 12% acrylamide resolve), alongside Dual Colored Protein Standards (Bio-Rad, Hercules, CA). The proteins in the samples were separated by electrophoresis (200 V constant, 1 hour). Samples were transferred to PVDF membranes by electrophoresis (100 V constant, 70 minutes) and then reacted with 1:500 Penta·His Antibody, BSA-free (monoclonal mouse anti-(H)_5_, Qiagen, Germantown, MD) Briefly, the membrane was blocked with 5% nonfat milk in TBS-T, and following incubation with primary antibodies, the membrane was washed 3 times with TBS-T. Bound primary antibodies were detected using a secondary Amersham ECL sheep anti-mouse horseradish peroxidase–conjugated antibody (species-specific whole antibody, NA931, GE Healthcare, Little Chalfont, Buckinghamshire, UK), at a dilution of 1:10,000. Detection was performed with SuperSignal West Pico Chemiluminescent Substrate kit (Thermo Scientific, Pierce, Rockford, IL) according to the manufacturer’s instructions, and then visualized using HyBlot CL Autoradiography Film (Denville Scientific, Holliston, MA).

**Yeast two-hybrid analysis***:* A commercially-available yeast two-hybrid system (ProQuest, Life Technologies) was employed that permitted plasmid constructing using GATEWAY technology. Plasmids were introduced into yeast strain MaV203 (MATα; *leu2-3,112; trp1-901; his3∆200; ade2-101; cyh2R; can1R; gal4∆; gal80∆; GAL1::lacZ; HIS3_UAS_GAL1::HIS3@LYS2; SPAL10 _UAS_GAL1::URA3*. Control plasmids with weak, moderate and strong interactions (LifeTechnologies) were assayed in parallel.

# Supporting information references

1. Miller J. A short course in bacterial genetics. Cold Spring Harbor, NY: Cold Spring Harbor Press; 1992.

2. Datsenko KA, Wanner BL. One-step inactivation of chromosomal genes in *Escherichia col*i K-12 using PCR products. Proc Natl Acad Sci U S A. 2000;97: 6640-6645.

3. Saka K, Tadenuma M, Nakade S, Tanaka N, Sugawara H, Nishikawa K, et al. A complete set of *Escherichia coli* open reading frames in mobile plasmids facilitating genetic studies. DNA Res 2005;12: 63-68.

4. Willetts NS, Clark AJ, Low B. Genetic location of certain mutations conferring recombination deficiency in *Escherichia coli*. J Bacteriol. 1969;97: 244-249.

5. Dower WJ, Miller JF, Ragsdale CW. High efficiency transformation of *E. coli* by high voltage electroporation. Nucleic Acids Research. 1988;16: 6127-6145.

6. Bachmann BJ. Pedigrees of some mutant strains of *Escherichia coli* K-12. Bacteriol Rev. 1972;36: 525-557.

7. Saveson CJ, Lovett ST. Enhanced deletion formation by aberrant DNA replication in *Escherichia coli.* Genetics. 1997;146: 457-470.

8. Cooper DL, Lovett ST. Toxicity and tolerance mechanisms for azidothymidine, a replication gap-promoting agent, in *Escherichia coli*. DNA Repair. 2011;10: 260-270.

9. Kolodner R, Fishel RA, Howard M. Genetic recombination of bacterial plasmid DNA: effect of RecF pathway mutations on plasmid recombination in *Escherichia coli.* 1985;163: 1060-1066.

10. Singer M, Baker TA, Schnitzler G, Deischel SM, Goel M, Dove W, et al. A collection of strains containing genetically linked alternating antibiotic resistance elements for genetic mapping of *Escherichia coli.* Microbiol Rev. 1989;53: 1-24.

11. Mount DW, Low KB, Edmiston SJ. Dominant mutations (*lex*) in *Escherichia coli* K-12 which affect radiation sensitivity and frequency of ultraviolet lght-induced mutations. J Bacteriol. 1972;112: 886-893.

12. Baba T, Ara T, Hasegawa M, Takai Y, Okumura Y, Baba M, et al. Construction of *Escherichia coli* K-12 in-frame, single-gene knockout mutants: the Keio collection. Mol Syst Biol. 2006;2:2006 0008.

13. Kitagawa M, Ara T, Arifuzzaman M, Ioka-Nakamichi T, Inamoto E, Toyonaga H, et al. Complete set of ORF clones of *Escherichia coli* ASKA library (a complete set of *E. col*i K-12 ORF archive): unique resources for biological research. DNA Res. 2005;12: 291-299.
