# Supplementary material for: Connecting Replication and Repair: YoaA, a Helicase-Related Protein, Promotes Azidothymidine Tolerance through Association with Chi, an Accessory Clamp Loader Protein
Source: PLoS Genet. 2015 Nov 6;11(11):e1005651. doi: 10.1371/journal.pgen.1005651 (PMC4636137; doi:10.1371/journal.pgen.1005651)
Supplement: S1 Table — Table S1A. Strains and plasmids. Table S1B. Oligonucleotides. Sequences are presented 5’ to 3’. (DOCX) [file pgen.1005651.s002.docx]

| **Supplemental Information Table S1. Strains, plasmids and oligonucleotides used in this study.**  **Table S1A** Strains and plasmids.**^[[1]](#footnote-1)^** | | | |
| --- | --- | --- | --- |
| **Strain** | **Relevant genotype** | **Source** |  |
| AB1157 | F- *argE3 hisG4 thr-1 leuB6 proA*- *gpt62*∆ *supE44 kdgK51 rfbD1 ara-14 lacY1 galK2 xyl-5 mtl-1 tsx-33 rpsL31* Rac*-* | [6] |  |
| MG1655 | F- *rph-1* | [6] |  |
| STL3000 | *mutD5* (AB1157 background) | [7] |  |
| STL7180 | *recA*::*cat* | [8] |  |
| RDK1309 | *ssb-113* (AB1157 background) | [9] |  |
| STL12838 | *ssb-113 malE::*Tn*10kan* (AB1157 background) | Km^R^ UV^S^ transductant RDK1309 [9] x P1 CAG12119 [10] |  |
| DM49 | *lexA3* (AB1157 background) | [11] |  |
| CAG12164 | *malF3089∆::*Tn*10* | [10] |  |
| STL17375 | *lexA3 malF3089∆*::Tn*10* | Tc^R^ UV^S^ transductant STL235 x P1 STL545 |  |
| STL17432 | *lexA3 malF3089∆*::Tn*10* | Tc^r^ UV^s^ transductant MG1655 x P1 STL17375 |  |
| STL9811 | *yoaA*∆FRT::*cat* | Cm^r^ λ Red recombinant |  |
| STL9813 | *yoaA*∆FRT | Cm^s^ derivative via pCP20 FLP mediated recombination |  |
| STL9479 | *dinG1*∆::*kan* | Km^r^ transductant MG1655 x P1 SY50 (H. Ohmori) |  |
| STL9814 | *yoaA*∆FRT *dinG1*∆::*kan* | Km^r^ transductant STL9813 x P1 STL5816 |  |
| STL18822 | *holC*∆FRT::*kan* | Km^R^ transductant MG1655 x P1 JW4216 Baba [12] |  |
| STL2107 | *holC102*::*cat* (AB1157 background) | [7] |  |
| STL19014 | *yoaA*∆FRT *holC*∆FRT::*kan* | Km^s^ transductant STL9813 x P1 STL18822 |  |
| STL10183 | *xthA*∆FRT::*cat* | [8] |  |
|  | | | |
| **Plasmid** | **Description** | **Source** |  |
| pNTR-Control | Ap, *araG* from mobile plasmid collection | National Institute of Genetics |  |
| pNTR-Control2 | Ap, *tfaE* from mobile plasmid collection | National Institute of Genetics |  |
| pNTR-HolC | Ap, *holC* | National Institute of Genetics |  |
| pNTR-HolC V117F | Ap, *holC* V117F | This work |  |
| pNTR-HolC Y131L | Ap, *holC* Y131L | This work |  |
| pNTR-HolC R128A | Ap, *holC* R128A | This work |  |
| pNTR-HolC F64A | Ap, *holC* F64A | This work |  |
| pNTR-YoaA | Ap, *yoaA* | National Institute of Genetics |  |
| pNTR-YoaA K51A | Ap, *yoaA* K51A | This work |  |
| pNTR-YoaA D225A | Ap, yoaA D225A | This work |  |
| pNTR-YoaA C168S | Ap, yoaA C168S | This work |  |
| pDONR221 | Km, Cm, vector for Gateway Cloning | LifeTechnologies |  |
| pDONR221-HolC | Km, *holC* N-terminal fusion construct | This work |  |
| pDONR221-YoaA | Km, *yoaA* N-terminal fusion construct | This work |  |
| pCA24N-HolC | Cm, *holC* ASKA 6 X HIS collection (pSTL386) | [13] |  |
| pCA24N-YoaA | Cm, *yoaA* ASKA 6 X HIS collection | Kitagawa 2006 |  |
| pET104.1-DEST | Ap, Cm, vector | LifeTechnologies pET104 BioEase Gateway Expression System |  |
| pET104.1-YoaA | Ap, yoaA-biotin binding domain (pSTL385) | This work |  |
| pDEST22 | Ap, GAL4 Activation Domain (AD) empty vector | LifeTechnologies |  |
| pDEST22-HolC | Ap, holC fused to GAL4 AD | This work |  |
| pDEST22-HolC F64A | Ap, holC F64A mutant allele fused to GAL4 AD | This work |  |
| pDEST22-YoaA | Ap, yoaA fused to GAL4 AD | This work |  |
| pDEST32- | Gm, GAL4 DNA Binding Domain (BD) empty vector | This work |  |
| pDEST32-HolC | Gm, holC fused to GAL4 BD | This work |  |
| pDEST32-YoaA | Gm, yoaA fused to GAL4 BD | This work |  |
| pDEST32-HolD | Gm, holD fused to GAL4 BD | This work |  |
| pPC97 | Gm, Y2H empty vector, no interaction control | LifeTechnologies |  |
| pPC86 | Ap, Y2H empty vector, no interaction control | LifeTechnologies |  |
| pPC97-RB | Gm, human RB Acc # M28419, amino acids 302-928 | LifeTechnologies |  |
| pPC86-E2F1 | Ap, human E2F1 Acc# M96577, amino acids 342-437 | LifeTechnologies |  |
| pPC97-CYH2S-dDP | Gm, Drosophila DP Acc# X79708, amino acids 1-377 | LifeTechnologies |  |
| pPC86-dE2F | Ap, Drosophila E2F Acc # U10184 amino acids 225-433 | LifeTechnologies |  |
| pPCL1 | Gm, encoding full-length GAL4, Acc # K10486 amino acids 1-881 | LifeTechnologies |  |

All strains are MG1655 derivatives unless noted otherwise as AB1157 derivatives.

| **Table S1B.** Oligonucleotides | | |
| --- | --- | --- |
| **Name** | **Use** | **Sequence (5’ to 3’)** |
| holC GWattB1 | GATEWAY cloning fusion HolC | GGGGACAAGTTTGTACAAAAAAGCAGGCTTCAAAAACGCGACGTTCTACCTTCT |
| holC GW attB2 | GATEWAY cloning fusion HolC | GGGGACCACTTTGTACAAGAAAGCTGGGTCCTGCTTTTCCCAGTGCTCGTA |
| holC F64A Forward | Quikchange HolC putative HolD binding residue | GCGTCCGGCAGAAAGCGCTGTTCCGCATAATTTAGCGGGAG |
| holCF64Aprimer2 | Quikchange HolC putative HolD binding residue | CTCCCGCTAAATTATGCGGAACAGCGCTTTCTGCCGGACGC |
| holCR128Aprimer1 | Quikchange HolC SSB binding residue | CTCTGAAACAACTGGCGGCCGAACGCTATAAAGCCTACC |
| holCR128Aprimer2 | Quikchange HolC SSB binding residue | GGTAGGCTTTATAGCGTTCGGCCGCCAGTTGTTTCAGAG |
| holCV117Fprimer1 | Quikchange HolC SSB binding residues | CCGCTTTCACAGAAGTGGTAGACTTCTTTCCTTATGAAGATTCTC |
| holCV117Fprimer2 | Quikchange HolC SSB binding residue | GAGAATCTTCATAAGGAAAGAAGTCTACCACTTCTGTGAAAGCGG |
| holCY131Lprimer1 | Quikchange HolC SSB binding residue | CTGGCGCGCGAACGCCTTAAAGCCTACCGCGTG |
| holCY131Lprimer2 | Quikchange HolC SSB binding residue | CACGCGGTAGGCTTTAAGGCGTTCGCGCGCCAG |
| HolDGWF1 | GATEWAY cloning fusion HolD | GGGGACAAGTTTGTACAAAAAAGCAGGCTTCACATCCCGACGAGACTGGC |
| holDGWF2 | GATEWAY cloning fusion HolD | GGGGACCACTTTGTACAAGAAAGCTGGGTCCTAGCTCGCCCTGGTTGCTGGC |
| LTB yoaA F gate fus | GATEWAY cloning fusion YoaA | GGGGACAAGTTTGTACAAAAAAGCAGGCTTCGTGACGGACGATTTTGCACCAGAC |
| LTB yoaA R gate | GATEWAY cloning fusion YoaA | GGGGACCACTTTGTACAAGAAAGCTGGGTCTTACCTGGAGGATGGTATCGCAAGGAA |
| FeSC168Sp1 | Quickchange of YoaA conserved Fe-S cluster | CAGCACCAACGACAACTCTCTTGGCAGCGACTG |
| FeSC168Sp2 | Quickchange of YoaA conserved Fe-S cluster | CAGTCGCTGCCAAGAGAGTTGTCGTTGGTGCTG |
| walaK51Ap1 | Quickchange of YoaA conserved Walker A box | GGAACCGGTACGGGCGCAACCTACGCTTACCTG |
| walaK51Ap2 | Quickchange of YoaA conserved Walker A box | CAGGTAAGCGTAGGTTGCGCCCGTACCGGTTCC |
| walbD225Ap1 | Quickchange of YoaA conserved Walker B box | GTCATGATCTTCGCCGAAGCCCACCAGCTACC |
| walbD225Ap2 | Quickchange of YoaA conserved Walker B box | GGTAGCTGGTGGGCTTCGGCGAAGATCATGAC |

1. [↑](#footnote-ref-1)
