# Supplementary material for: Connecting Replication and Repair: YoaA, a Helicase-Related Protein, Promotes Azidothymidine Tolerance through Association with Chi, an Accessory Clamp Loader Protein
Source: PLoS Genet. 2015 Nov 6;11(11):e1005651. doi: 10.1371/journal.pgen.1005651 (PMC4636137; doi:10.1371/journal.pgen.1005651)
Supplement: S2 Table — Relative plating efficiency on LB medium containing the indicated AZT concentrations relative to that on LB medium without AZT. (DOCX) [file pgen.1005651.s003.docx]

Supplemental Information **Table S2: Fractional survival of *holC* and *holC yoaA* strains expressing mobile plasmids on medium containing AZT.** Relative plating efficiency on LB medium containing the indicated AZT concentrations relative to that on LB medium without AZT.

| Strain | Plasmid | 12.5 ng/mL AZT | 25 ng/mL AZT |
| --- | --- | --- | --- |
| *holC* | pNTR-Control | 3.5 x 10^-4^ | 2.3 x 10^-5^ |
| *holC* | pNTR-HolC | 7.3 x 10^-1^ | 4.3 x 10^-1^ |
| *holC* | pNTR-YoaA | 3.2 x 10^-4^ | 7.4 x 10^-5^ |
| *holC yoaA* | pNTR-Control | 8.0 x 10^-4^ | 1.0 x 10^-4^ |
| *holC yoaA* | pNTR-HolC | 4.1 x 10^-3^ | 1.2 x 10^-3^ |
| *holC yoaA* | pNTR-YoaA | 6.3 x 10^-4^ | 6.8 x 10^-5^ |
