# Supplementary figures and images for: Connecting Replication and Repair: YoaA, a Helicase-Related Protein, Promotes Azidothymidine Tolerance through Association with Chi, an Accessory Clamp Loader Protein
Source: PLoS Genet. 2015 Nov 6;11(11):e1005651. doi: 10.1371/journal.pgen.1005651 (PMC4636137; doi:10.1371/journal.pgen.1005651)

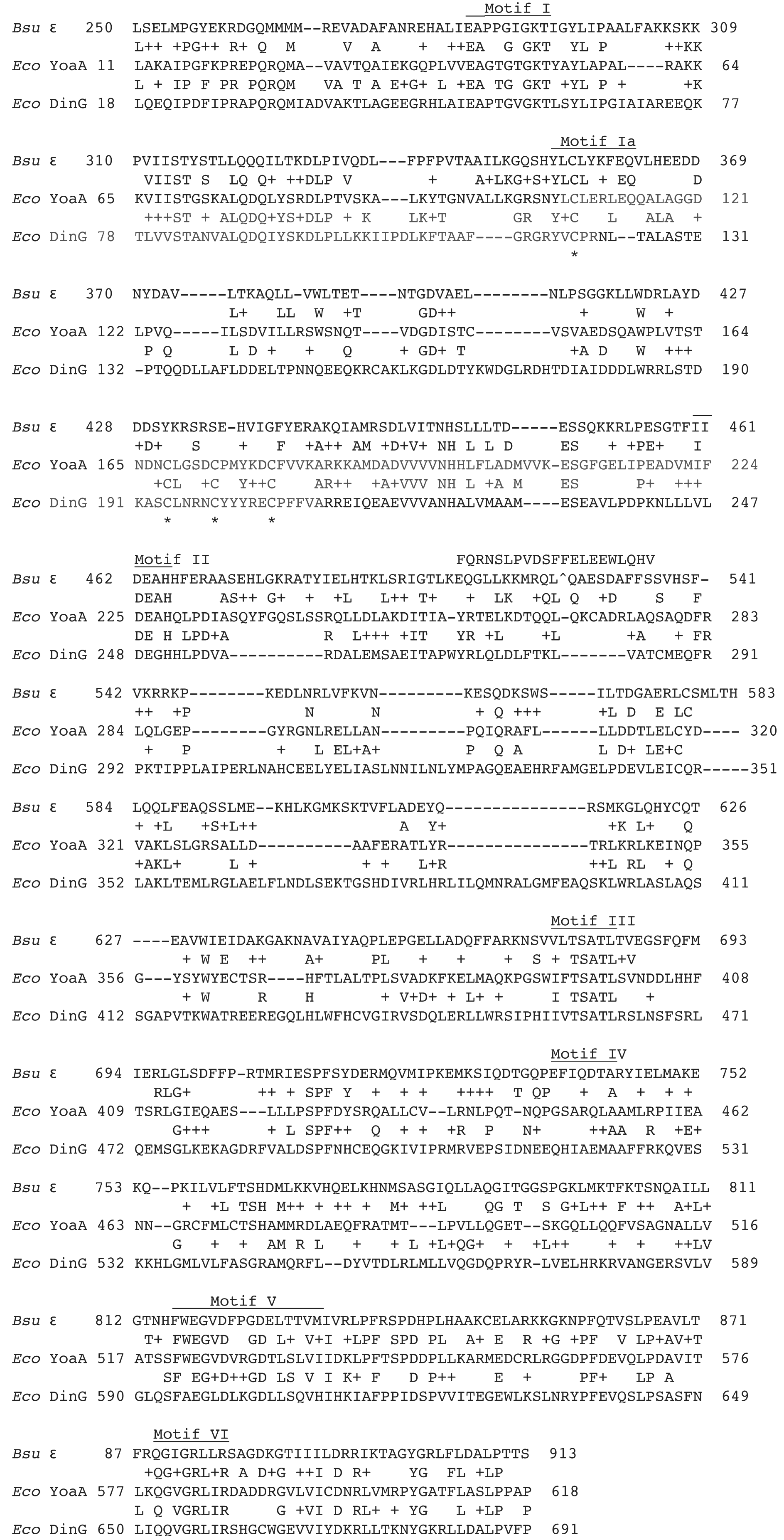

Supplement: S2 Fig — Above the alignment are indicated the conserved helicase motifs and below, with asterisks, the Fe-S coordination residues for DinG. (TIF) [file pgen.1005651.s005.tif]
